# Supplementary material for: The effects of exercise on FGF21 in adults: a systematic review and meta-analysis
Source: PeerJ. 2024 Jun 25;12:e17615. doi: 10.7717/peerj.17615 (PMC11212618; doi:10.7717/peerj.17615)
Supplement: Supplemental Information 4 [file peerj-12-17615-s004.docx]

**Different types of exercise training on FGF21 in adults: a meta-analysis and systematic review**

**P:** adults

**I:** any types exercise training regimen

**C:** sedentary or light intensity physical activity participants

**O:** FGF21 levels

**S:** RCT

Databases (1129): **Pubmed, Cochrane Library, web of science**

**Attention:**

As the reviewer`s advice, we improved the search strategy. Due to variations in data caused by different search times, we have excluded literature after July 2023. The number in the parentheses”()” represents the actual quantity of literature and the picture shown the quantity of April 2024.

**Pubmed(605)**

(FGF21 OR FGF-21 OR fibroblast growth factor 21) AND (training OR exercise OR exercise training OR physical activity)


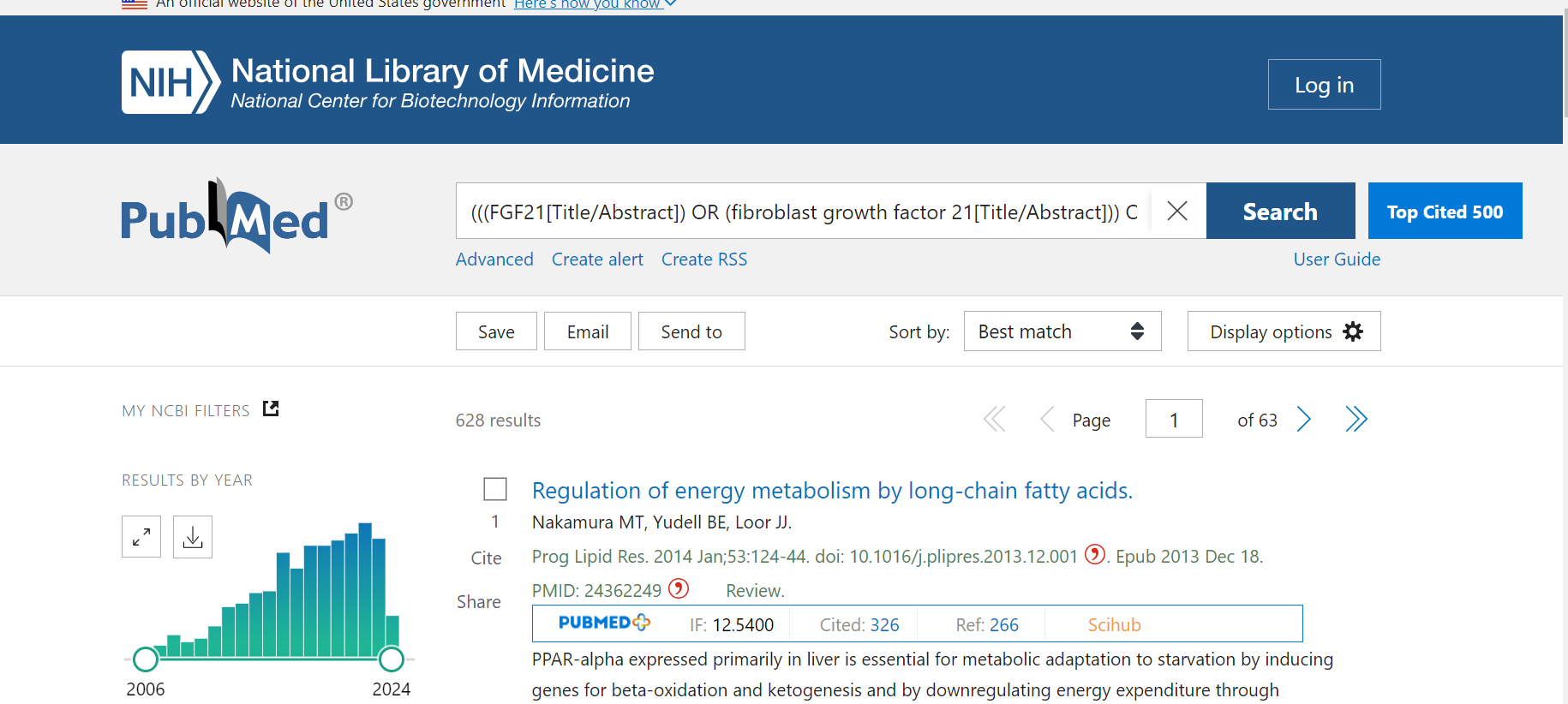


**Cochrane Library****(163)**

(FGF21 OR FGF-21 OR fibroblast growth factor 21) AND (training OR exercise OR exercise training OR physical activity)


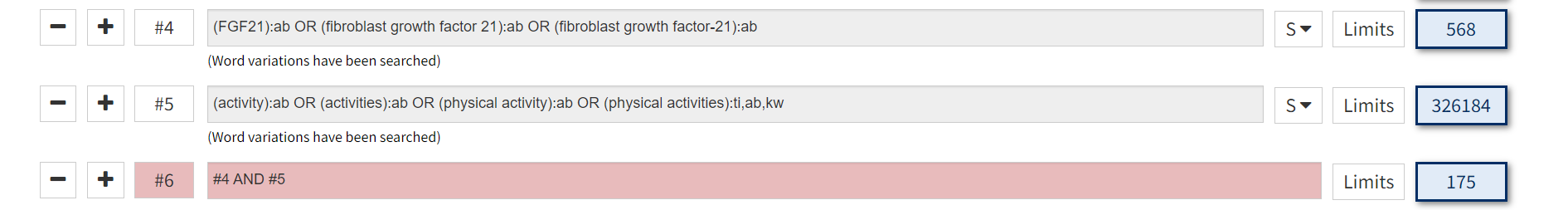


**Web of Science (1131)**

(FGF21 OR FGF-21 OR fibroblast growth factor 21) AND (training OR exercise OR exercise training OR physical activity)

**
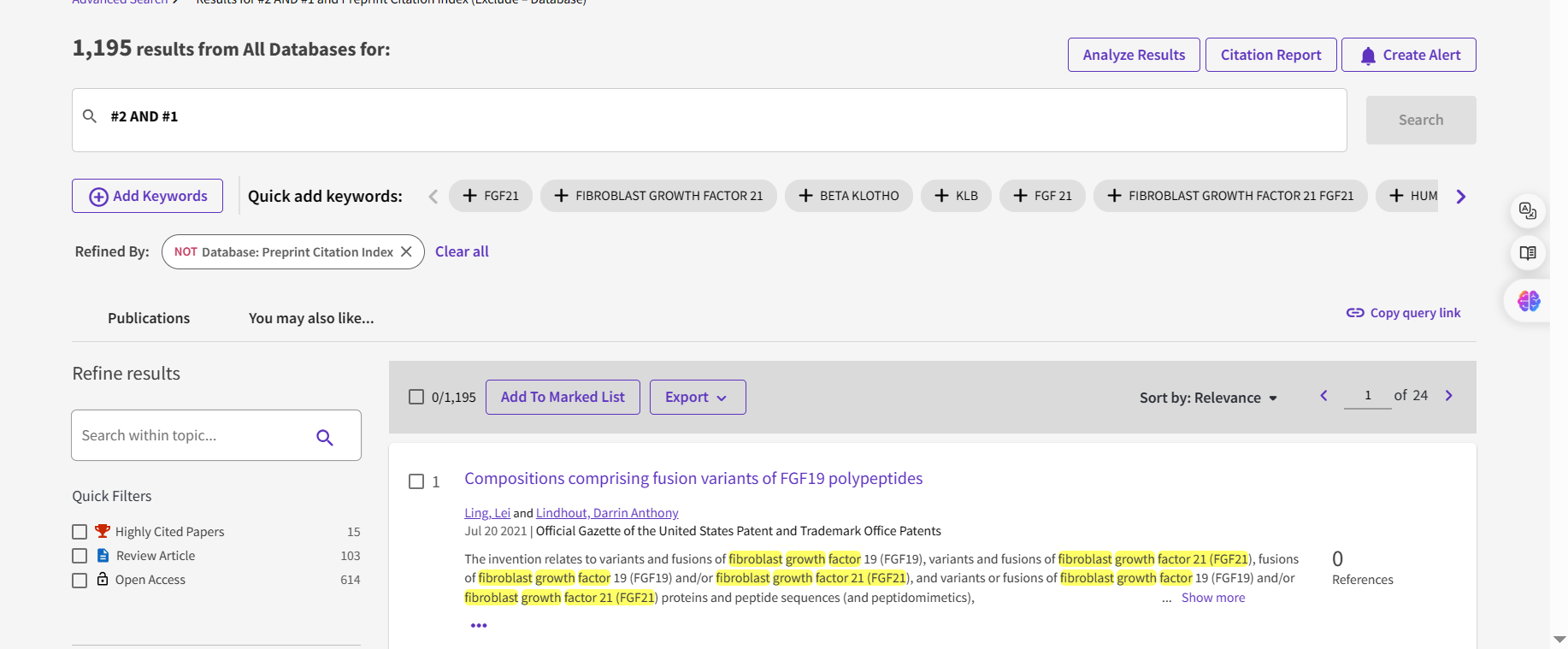
**

The sensitivity analysis result of all the available studies(18) on this manuscript, it shown all the studies are usable and the result effect is robustness.


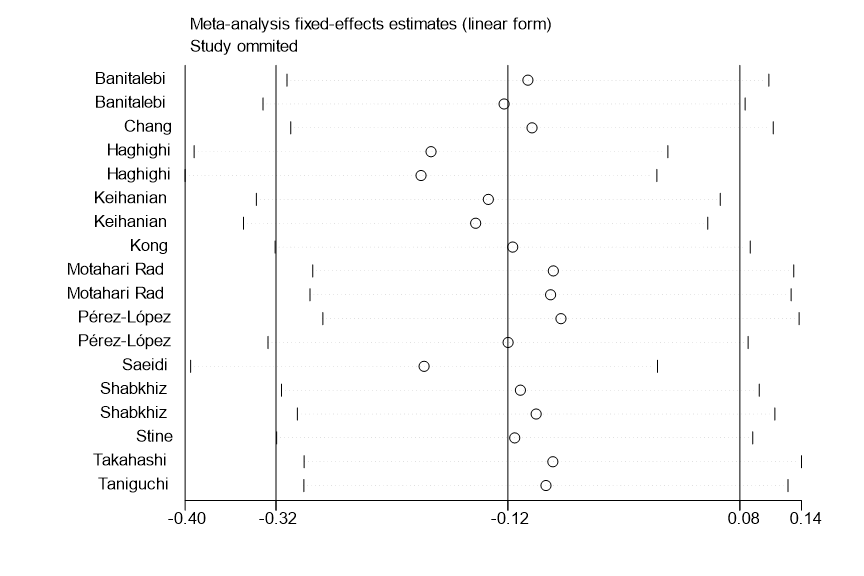


| **Abbreviation list** | |
| --- | --- |
| Abbreviation | Full name |
| FGF21 | fibroblast growth factor 21 |
| T2D | type 2 diabetes |
| NAFLD | non-alcoholic fatty liver disease |
| NASH | non-alcoholic hepatitis |
| CNKI | Chinese National Knowledge Infrastructure |
| SMD | STD mean difference |
| CI | confidence interval |
| FGF | fibroblast growth factor |
| KLB | beta-klotho |
| WAT | white adipose tissue |
| BAT | browning adipose tissue |
| AMPK | AMP-activated protein kinase |
| MVPA | moderate to vigorous intensity physical activity |
| PICOS | population,intervention,comparison,outcome,study design |
| BMI | body mass index |
| HIIT | high intensity interval training |
| VO2max | maximum oxygen uptake |
| RM | maximum repetition |
| FBS | fasting blood sugar |
| LDL | low density lipoportein |
| FFAs | free fat acid |
| PPARα | peroxisome porliferators activated receptor α |
| GDF15 | growth differentiation factor 15 |
| IL-6 | interleukin 6 |
| FGFR1c | fibroblast growth factor receptor 1c |
