# Supplementary material for: The effects of exercise on FGF21 in adults: a systematic review and meta-analysis
Source: PeerJ. 2024 Jun 25;12:e17615. doi: 10.7717/peerj.17615 (PMC11212618; doi:10.7717/peerj.17615)
Supplement: Supplemental Information 3 [file peerj-12-17615-s003.docx]

Systematic Review and/or Meta-Analysis Rational

Dear editor/reviewer,

In this paper, we main reported that exercise could improve the FGF21 levels in adults with obese, T2D, aging, NASH etc.. The available studies had analysis different exercise types, duration(10 weeks), and intensity(80% 1RM or VO2 peak). The results shown the concurrent exercise(aerobic + resistance) type, resistance exercise and duration≥10 weeks decrease FGF21 level significant. Moreover, FGF21 is key factor in metabolic homeostasis, so this is important for this field. There were studies researched on exercise on FGF21, but not integrity and different perspectives. There is a systematic review about aerobic exercise on FGF21 in human and animal which focus on the molecular mechanism not exercise itself. Another related meta-analysis did the same subgroup analysis, but the group is not accuracy and the final result is different. So we did the meta-analysis more properly, exercise type, intensity and the term, which more focus on exercise training.In addition, add several key studies to discuss this, like a update.

According to all the reasons, our team decided do this review.

Sincerely

Chuannan liu
